# Adaptive Reinforcement Learning-Based Framework for Energy-Efficient Task Offloading in a Fog–Cloud Environment

**DOI:** 10.3390/s25247516

**Published:** 2025-12-10

**Authors:** Branka Mikavica, Aleksandra Kostic-Ljubisavljevic

**Affiliations:** Faculty of Transport and Traffic Engineering, University of Belgrade, Vojvode Stepe 305, 11010 Belgrade, Serbia; a.kostic@sf.bg.ac.rs

**Keywords:** fog computing, Internet of Things, task offloading, reinforcement learning, resource allocation, energy efficiency, delay optimization

## Abstract

Ever-increasing computational demand introduced by the expanding scale of Internet of Things (IoT) devices poses significant concerns in terms of energy consumption in a fog–cloud environment. Due to the limited resources of IoT devices, energy-efficient task offloading becomes even more challenging for time-sensitive tasks. In this paper, we propose a reinforcement learning-based framework, namely Adaptive Q-learning-based Energy-aware Task Offloading (AQETO), that dynamically manages the energy consumption of fog nodes in a fog–cloud network. Concurrently, it considers IoT task delay tolerance and allocates computational resources while satisfying deadline requirements. The proposed approach dynamically determines energy states of each fog node using Q-learning depending on workload fluctuations. Moreover, AQETO prioritizes allocation of the most urgent tasks to minimize delays. Extensive experiments demonstrate the effectiveness of AQETO in terms of the minimization of fog node energy consumption and delay and the maximization of system efficiency.

## 1. Introduction

The proliferation of Internet of Things (IoT) applications and rapid development of cloud-based technologies lead to the continuous generation of massive amounts of data and substantially increase demand for computation resources [1]. In general, IoT devices are resource-constrained, thus disabling local execution of computation tasks. Traditional cloud computing systems, on the other hand, provide robust computing resources that can manage enormous amounts of data. However, due to the centralized cloud architecture and deadline-sensitive nature of IoT applications, including smart home, Intelligent Transportation Systems (ITS), manufacturing, drone navigation, smart healthcare, and augmented reality, traditional cloud computing fails to meet strict delay requirements and real-time response [2]. Fog–cloud computing emerged as a promising solution for these challenges [3,4]. The fog–cloud architecture comprises three layers: IoT devices, a fog layer, and a cloud layer. Typically, in the intermediate fog layer, fog servers are placed in the network edge, bringing computation resources in the close vicinity of IoT devices. Execution of computation tasks at the network edge reduces delays and improves computation efficiency. However, the introduction of a fog layer raises additional issues concerning task execution location, i.e., whether tasks are to be offloaded to the fog or cloud [5]. The offloading decision usually includes optimization under different constraints in terms of delay, energy consumption, and resource allocation. The development of an efficient task offloading strategy in a fog–cloud network is highly challenging due to the heterogeneity of fog nodes, with frequent fluctuations in nodes’ connectivity, resource availability, and task arrival patterns [6].

Compared to the traditional cloud systems, fog computing significantly reduces delay and energy consumption by processing data at the network edge, which is highly beneficial for IoT applications such as intelligent logistics and real-time process monitoring in industrial environments [7]. Ensuring energy-efficient task offloading remains a critical concern in fog–cloud environments. An efficient task offloading technique must encompass the strategic allocation and the distribution of IoT tasks among various devices, fog nodes, and remote cloud data centers based on the energy consumption considerations. One of the promising energy-saving strategies in fog computing is selectively powering off unused resources [8]. The magnitude of energy savings by implementing the switching on/off technique depends on the various factors, including fog network scale, the frequency of resource switching, and the energy constraints in the environment [9]. An effective switching on/off technique can optimize energy consumption and extend the operational life of fog nodes. However, this technique also introduces a potential trade-off in the form of delay due to the resource unavailability during transition [10].

Besides delay and energy consumption, the task offloading problem in fog–cloud environments inherently involves effective resource allocation, which becomes even more critical when applying energy-saving strategies such as the switching on/off technique [1,9]. Machine learning is often referred to as a powerful technique for energy-efficient resource allocation in fog–cloud environments [11]. Among them, reinforcement learning [12] and deep learning [13] techniques are most commonly used in task offloading optimization. Reinforcement learning approaches are based on learning the best offloading policies under various constraints, including workload, delay, and energy consumption. A major limitation of these approaches is scalability issues in large-scale fog environments. Deep learning-based approaches solve the issue of scalability; however, they introduce high computation complexity and require large data sets, thus struggling with real-time adaptability and hindering their effectiveness in highly dynamic fog–cloud environments.

Motivated by these challenges, in this paper, we propose an efficient reinforcement learning-based framework to dynamically manage energy consumption of fog nodes in a fog–cloud network by minimizing fog idle resources depending on workload fluctuations while minimizing delay in the task offloading process. We adopt a Q-learning approach, which is well suited for environments with a tractable decision space, enabling the framework to adapt incrementally to workload changes. To the best of our knowledge, this is the first paper that considers adaptive idle resource reduction together with energy state transition management, achieving an effective balance between energy savings and demand satisfaction for time-sensitive IoT tasks.

The main contributions of this paper can be summarized as follows:

(i) We propose a novel, reinforcement learning-based framework, Adaptive Q-learning-based Energy-aware Task Offloading (AQETO), to jointly minimize the energy consumption of fog nodes and task offloading delay in a fog–cloud network;
(ii) AQETO incorporates a priority mechanism that favors urgent tasks to ensure timely task execution under strict deadline requirements;
(iii) AQETO dynamically determines the energy states of fog nodes and optimizes transitions between active and sleep modes, thus achieving a balanced trade-off between energy efficiency and demand satisfaction;
(iv) Unlike previous studies, AQETO considers delays and additional energy consumption due to the transition between the energy states, thus modeling resource unavailability and preventing excessive switching between the energy states.
(v) Extensive simulations are conducted to validate the performance and effectiveness of AQETO under dynamic workload fluctuations.

The rest of the paper is organized as follows. After introductory remarks, Section 2 provides a literature review. In Section 3, the system model and problem formulation are presented. The proposed AQETO framework is described in Section 4. Numerical results and performance evaluation are provided in Section 5, and discussed in Section 6. Finally, concluding remarks and future research directions are presented in Section 7.

## 2. Related Works

The task offloading problem in fog–cloud environments often includes balancing diverse parameters. Joint optimization of energy consumption, resource allocation, and delay is a highly challenging issue. In [14], an omnidirectional offloading algorithm based on the Modified Simulated Annealing (MSA) is proposed to reduce energy consumption in a federated cloud–edge–fog system. The results show a reduction in energy consumption of 11–27% when compared with other heuristic approaches. The Optimal Energy-efficient Resource Allocation (OEeRA) based on Minimal Cost Resource Allocation (MCRA) and Fault Identification and Rectification (FIR) algorithms are developed in [15] to improve the network energy efficiency and task offloading in IoT-fog environments. In [16], an adaptive approach leveraging the Multi-Armed Bandit (MAB) method, Energy-Delay Aware Binary Task Offloading Strategy (EDABTOS), is proposed to dynamically optimize task execution and resource allocation in fog-enabled IoT systems. Reported improvements are a 16.21% reduction in delay and 3.19% improvements in energy efficiency. A Deadline-aware Energy and Latency-optimized Task Offloading and resource allocation (DELTA) strategy is formulated as a multi-objective Mixed Integer Programming (MIP) problem in [17], with the aim to minimize the energy consumption of user equipment and the latency of applications while concurrently satisfying deadline and dependency constraints. The results show that DELTA outperforms all benchmark approaches by all relevant parameters.

Due to its capability to learn even when minimal information about the parameters is available, reinforcement learning is one of the most promising machine learning techniques for solving offloading issues and resource management in fog environments [18,19,20,21,22,23,24] and Mobile Edge Computing (MEC) [25,26,27,28,29]. A reinforcement learning-based model is proposed in [30] to predict the resource availability on the edge device and determine the optimal task offloading location. The proposed model achieves efficient resource utilization and reduces resource idle time on edge devices. In [31], a joint radio and computation resource allocation problem in fog-assisted mobile IoT networks is formulated as an integer non-linear problem, and an online reinforcement learning-based resource allocation algorithm is proposed. The aim of the proposed model is to improve the task delay of all tasks. Extensive simulations demonstrate its effectiveness over the baseline algorithms. In [32], an offloading and resource allocation algorithm based on Soft Actor–Critic (SAC) and federated Self-Supervised Learning (SSL) is proposed to reduce energy consumption and improve offloading efficiency in Vehicle Edge Computing (VEC) environment. This algorithm offloads partial tasks to Road Side Units (RSUs) and adjusts transmission power, CPU frequency, and task assignment rations, thus balancing local and RSU-based training.

Among reinforcement learning approaches, Q-learning is also often used for resource allocation and system performance improvements in fog computing [3,33,34,35,36,37], MEC [38], and edge computing environments [39,40,41,42].

The problem of efficient task offloading and resource allocation in IoT systems is addressed in [43]. A reinforcement learning-based framework, Group Relative Policy Optimization (GRPO), is proposed to reduce service delay and energy consumption. GRPO is designed as a Markov Decision Process (MDP) and considers several task features, including device mobility, status of the network, edge resource availability, and task completion deadlines. The results show that the proposed framework can successfully balance real-time changes in the task demand, mobility patterns, and available resources. A Decision Tree Empowered Reinforcement Learning (DTRL) technique is used in [44], to optimize the problem of task offloading and resource allocation for IoT applications. Simulation results prove that the proposed approach outperforms the state-of-the-art approaches in terms of delay, energy consumption, waiting time, task acceptance ratio, and service cost.

A joint task offloading and resource allocation algorithm based on Deep Q-Network (DQN) in task-dependent multi-access edge computing systems is proposed in [45], to minimize the weighted sum of the long-term task execution delay and energy consumption of IoT devices, with the maximum tolerable delay as a critical constraint. To accurately model task dependencies in the IoT task offloading process, in [2], a dynamic strategy based on an improved Double Deep Q-Network (DDQN), namely the Adaptive Dynamic Cloud–fog Computing Offloading Method for Complex Dependency Tasks (CADCO), is proposed. Practical application of CADCO is validated through both theoretical and experimental analysis, while the results demonstrate the effectiveness in terms of improved resource utilization and reduced task delay and energy consumption.

In [46], a Multi-Agent Deep Reinforcement Learning (MADRL)-based algorithm is proposed to optimize Quality of Experience (QoE), defined as a weighted sum of improvements in energy efficiency and delay in the context of cloud–edge–end collaboration. The results show that the proposed method outperforms baseline methods. Another multi-agent reinforcement learning-based framework, namely Multi-Agent Twin Delayed Deep Deterministic Policy Gradient for Task Offloading and Resource Allocation (MATD3-TORA), is proposed in [47], to optimize task offloading and resource allocation in Unmanned Aerial Vehicle (UAV)-assisted Mobile Edge Computing networks. MATD3-TORA jointly optimizes task processing delay and UAV energy consumption. The results demonstrate the effectiveness in terms of mobility–energy tradeoffs, distributed decision-making, and real-time resource allocation. In [48], a Multi-Agent Reinforcement Learning (MARL)-based scheduling framework dynamically allocates tasks based on environmental variations and workload fluctuations with the aim to ensure a balance between energy efficiency and system performance.

An integration of Graph Reinforcement Learning (GRL) with Asynchronous Federated Learning (AFL) is formulated as a framework (AFedGRL) in [6], to achieve efficient resource allocation and reduce task response delay in a fog computing environment. GRL is also used in [49] to effectively capture the dynamics in the offloading decision-making in a fog–edge continuum. Such a stable matching approach improves energy efficiency by 44% and achieves a task completion rate 3% to 12% higher than the benchmark algorithms. A GRL-based task offloading algorithm in an MEC environment and device-to-device (D2D) communication is proposed in [50], where a Graph Neural Network (GNN) is used to model the collaborative task offloading. The proposed algorithm introduces penalties when the tasks’ deadlines are exceeded. The results indicate a reduction in energy consumption by approximately 20% and improvements in task completion rates and load balancing.

All these studies on reinforcement learning-based optimization in fog computing environments provide valuable contributions to resource allocation and performance improvements in the task offloading process. However, none of them address management of energy states of fog nodes. Moreover, we aim to jointly minimize delay and energy consumption of fog nodes under deadline requirements while maximizing system efficiency.

## 3. System Model and Problem Formulation

In this section, we describe the architecture and formulate the task offloading problem in a fog–cloud environment.

### 3.1. System Model

**Figure 1:**
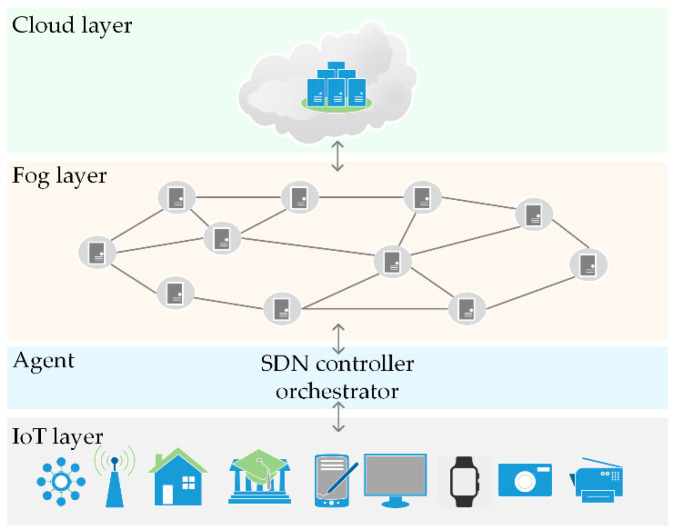
System architecture.

We consider a fog–cloud scenario comprising a layered architecture, as shown in Figure 1.

The IoT devices layer includes a wide variety of sensor devices and smart devices used in smart homes, healthcare, Intelligent Transportation Systems (ITS), virtual and augmented reality, etc. These devices generate a vast amount of heterogeneous data and pose severe Quality of Service (QoS) requirements. The diverse requests for IoT task execution occupy substantial computation and storage capacities. Due to the resource-constrained capability of IoT devices and considerable energy consumption, IoT tasks are being offloaded to the nearby fog nodes or remote cloud data center.

The fog layer consists of arbitrarily distributed fog nodes that constitute a fog network. We model the fog network as a graph, where all fog nodes are represented as nodes in the network, while edges established between the nodes represent communication links. Due to virtualization, computing and storage capabilities of fog nodes can be segmented into discrete unit allocations, Computing Resource Blocks (CRBs) [51].

The cloud layer is represented as a remote cloud data center. We assume that tasks are offloaded to the cloud data center if and only if the task cannot be processed in the fog layer due to the computing resource unavailability. For simplicity, we assume that the computational capacity of the cloud data center is large enough to provide offloading to any number of tasks.

The proposed reinforcement learning-based task offloading framework introduces an agent that can be conceptualized as a Software Defined Network (SDN) controller empowered by a fog node orchestrator [20,52]. The role of the SDN controller is to dynamically manage energy states of fog nodes in the network depending on the current workload, while the fog node orchestrator is responsible for the IoT task assignment based on the fog nodes’ availability and performance constraints, offloading tasks to the cloud when necessary, i.e., when no fog node has sufficient available resources to execute the task.

### 3.2. Problem Formulation

We divide the observed time horizon into time slots of equal length Δt. Let set of all requests for task offloading in a single time slot be $RQ=\left\{rq_{1},\;rq_{2},\ldots ,rq_{n}\right\}$. Each request for task offloading $rq_{i},\;i\in \left[1,n\right]$ can be described as a tuple $\left(d_{i},\sigma_{i}\right)$, where $d_{i}$ and $\sigma_{i}$ denote the task’s maximum delay tolerance and size, respectively. In this paper, we use the terms task and request for task offloading interchangeably. Without loss of generality, we assume that all requests are generated at the beginning of each time slot and can be executed within a single time slot. We also assume that tasks are atomic and independent [5,51]. To ensure delay minimization, we prioritize task offloading to fog nodes. Due to the atomicity, each task can be offloaded to a single fog node or to the cloud data center.

The set of all fog nodes is denoted as $F=\left\{f_{1},\;f_{2},\ldots ,f_{m}\right\}$. The computing capacity of a fog node $f_{j},\;j\in \left[1,m\right]$ is denoted by $\theta_{j}$ and expressed in CRBs. We assume that each fog node can process multiple tasks concurrently in each time slot. Computation energy consumption of each fog node depends on the workload to be executed. However, a fog node consumes substantial energy even in the idle state. A promising solution for reducing energy consumption is to reduce the number of idle nodes and switch them into low-power sleep states [53]. In accordance with this, in this paper we assume that a fog node can be in the wake or sleep state. Fog nodes in a wake state are either active (process tasks) or idle (do not process tasks but can be instantly activated when needed). Fog nodes in a sleep state are not fully deactivated, but energy-consuming modules are switched off to reduce energy consumption. However, the transition between states introduces additional energy consumption and delays due to the fog node’s unavailability during transition. We denote the set of wake, active, idle, and sleep fog nodes as W, AC, I and SL, respectively. The set of fog nodes that are transitioning to sleep state is denoted by $T_{s}$, while the set of fog nodes transitioning from sleep to wake state is denoted by $T_{w}$. We assume that only idle nodes can be transitioned to a sleep state. It applies $F=W\cup T_{w}\cup T_{s}\cup SL$ and W=AC∪I. To ensure that a fog node can be in a single energy state in a time slot, it applies AC∩I=∅, $AC\cap T_{w}=\emptyset$, $AC\cap T_{s}=\emptyset$, AC∩S=∅, $I\cap T_{w}=\emptyset$, $I\cap T_{s}=\emptyset$, I∩SL=∅, $T_{w}\cap T_{s}=\emptyset$, $T_{w}\cap SL=\emptyset$ and $T_{s}\cap SL=\emptyset$.

Let $l_{ij}$ be the shortest delay from the IoT device that generates a request for task offloading $rq_{i}$ to the fog node $f_{j}$, and let $l_{ic}$ be the shortest delay to the cloud data center. We determine a feasibility set for each request denoted by $\Phi_{i}$. A fog node or cloud data center is considered feasible for request $rq_{i}$ if the deadline requirement is satisfied.

Our objective is to minimize the computation energy consumption in a given fog–cloud network. Specifically, we focus exclusively on the computation energy consumption of the fog layer, since the vast majority of the tasks are being offloaded to this layer. Jointly, we aim to minimize delay while satisfying deadline requirements in IoT task offloading. Due to the assumption that each task can be executed within a single time slot, the processing time is negligible relative to the slot duration, and thus we consider only the communication delay component.

Therefore, we formulate the task offloading optimization problem as follows:

(1) $$\min\sum_{f_{j}\in AC}e_{j,active}+\sum_{f_{j}\in I}e_{idle}+\sum_{f_{j}\in T_{s}}e_{ws}+\sum_{f_{j}\in T_{w}}e_{sw}+\sum_{f_{j}\in SL}e_{sleep}$$

(2) $$\min\sum_{r_{i}}\sum_{f_{j}}x_{ij}\cdot l_{ij}+\sum_{r_{i}}y_{i}\cdot l_{ic}$$

subject to

(3) $$e_{j,active}=e_{idle}+\left[\left(e_{peak}-e_{idle}\right)\cdot U_{j}\right],\;\forall f_{j}\in F$$

(4) $$x_{ij}=\left\{\begin{array}{c} 1,\;if\;rq_{i}\;is\;assigned\;to\;f_{j}\\ 0,\;\;otherwise \end{array}\right.$$

(5) $$y_{i}=\left\{\begin{array}{c} 1,\;if\;rq_{i}\;is\;assigned\;to\;c\\ 0,\;\;otherwise \end{array}\right.$$

(6) $$U_{j}=\frac{\omega_{j}+\sum_{r_{i}}x_{ij}\cdot d_{i}}{\theta_{j}},\;\forall f_{j}\in F$$

(7) $$\sum_{f_{j}\in \Phi_{i}}x_{ij}+y_{i}\leq 1,\;\forall rq_{i}\in RQ,\;c\in \Phi_{i}$$

(8) $$l_{ij}\leq d_{i},\;\forall f_{j}\in \Phi_{i}$$

(9) $$l_{ic}\leq d_{i},\;\forall rq_{i}\in RQ,\;c\in \Phi_{i}$$

(10) $$\omega_{j}+\sum_{r_{i}}x_{ij}\cdot d_{i}\leq \theta_{j},\;\forall f_{j}\in \Phi_{i}$$

The objective functions (1) and (2) serve to minimize the energy consumption of fog nodes and minimize delay, respectively. Total energy consumption considers energy consumed by fog nodes in all respective energy states. Energy consumption of each active fog node that serves requests for task offloading is denoted as $e_{j,active}$ and determined by (3), where energy consumed at a maximum load is denoted by $e_{peak}$. Energy that a fog node consumes in idle and sleep states is denoted as $e_{idle}$ and $e_{sleep}$, respectively. Energy consumption for transitioning from wake to sleep and from sleep to wake state is denoted by $e_{ws}$ and $e_{sw}$, respectively. In (2), a binary parameter $x_{ij}$ denotes whether a task is allocated to the fog node or not. This parameter is constrained by (4). Similarly, in (2), a binary parameter $y_{i}$ determines whether a task is assigned to the cloud and is constrained by (5). The parameter $U_{j}$ in (3) denotes the fog node’s resource utilization and can be calculated by (6). In (6), the amount of previously occupied resources is denoted by $\omega_{j}$. Constraint (7) ensures task atomicity and allows task assignment to a single fog node or a cloud data center. Constraints (8) and (9) ensure that the communication delay must be less than or equal to the maximum delay tolerance. Therefore, a task can be assigned to a fog node or cloud if feasible. Constraints (10) bound the total amount of allocated computing resources of fog nodes by the amount of their maximum capacity.

Table 1 provides a comprehensive summary of notations used and corresponding descriptions.

## 4. The Proposed AQETO Framework

In this section, we introduce the proposed AQETO, a Q-learning-based framework for energy-aware task offloading in a fog–cloud environment. In the first phase of AQETO, the SDN controller has the role of the agent that performs offline learning to obtain near-optimal policy for the determination of the fog nodes’ energy states. The agent aims to jointly satisfy demand for computing resources of fog nodes and minimize energy consumption by reducing the number of wake nodes whenever possible. This approach remains feasible in our setting because both the state and action spaces are discretized into a small number of levels, which keeps the Q-table manageable and ensures efficient learning. Once the optimal policy for node distribution in a given fog–cloud environment is obtained, the fog node orchestrator performs task assignment to wake nodes aiming to minimize delay.

### 4.1. Q-Learning-Based Energy Consumption Management

**Figure 2:**
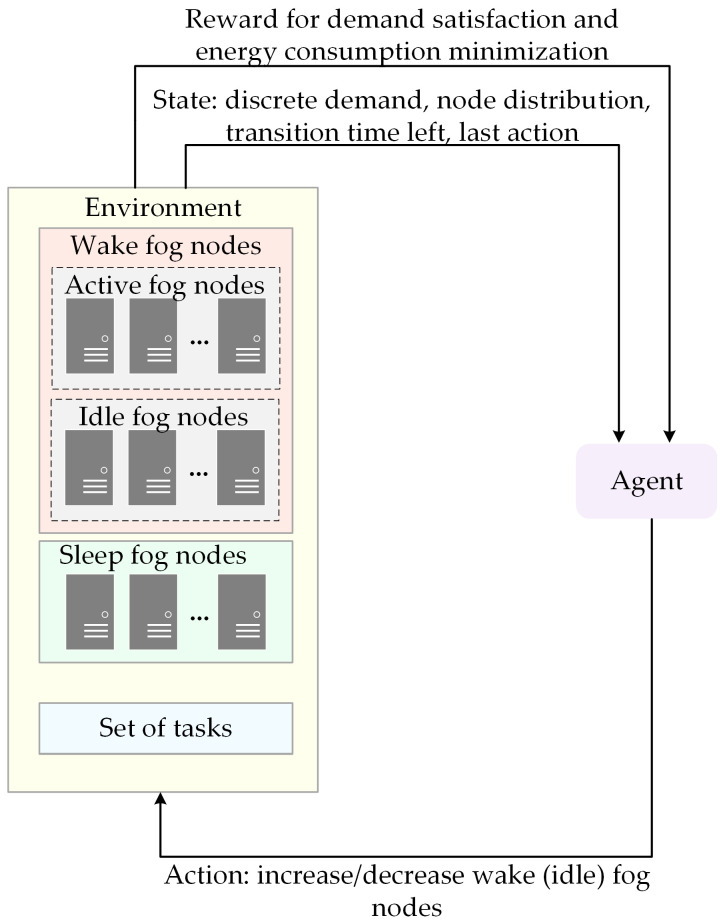
RL agent’s interaction with the environment in AQETO framework.

For every generated workload, the agent calculates the available computing capacity of currently wake nodes, determines the minimum required number of wake fog nodes that can satisfy total demand, and adjusts the number of wake nodes if needed, as shown in Figure 2.

When the demand exceeds the currently available computing resources of wake nodes, the agent tries to transition fog nodes from sleep (if there are any) to the wake state as a response to the given workload. On the other hand, when the currently available computing resources of fog nodes are underutilized, the agent reduces the number of idle nodes and transitions them to a sleep state to reduce energy consumption. To ensure fair transitioning logic, the agent transitions the least often transitioned nodes. However, fog nodes cannot be immediately switched between states. During transition, the transitioning fog node is unavailable. The transition durations from wake to sleep and sleep to wake state are denoted by $\tau_{s}$ and $\tau_{w}$, respectively. It applies $\tau_{w}>\tau_{s}$. Moreover, transitions introduce additional energy consumption, so frequent transitions are not preferable. Therefore, the agent must allocate resources to the current workload and minimize energy consumption with the minimum number of transitions between states.

Our goal is decision-making for the energy state of each fog node. Therefore, to ensure robustness, we set the state space using critical factors that encapsulate the environmental conditions in time slot t, denoted as

(11) $$S_{t}=\left[D_{t},WN_{t},IN_{t},SN_{t},TT_{t},LA_{t}\right],$$

where $D_{t}$ is the discrete value of total demand for fog nodes computing resources. The set of fog nodes in wake, idle, and sleep energy states are denoted as $WN_{t}$, $IN_{t}$ and $SN_{t}$, respectively. To improve convergence, we express $D_{t}$, $WN_{t}$, $IN_{t}$ and $SN_{t}$ as the percent of full computing capacity of the fog layer. Moreover, $D_{t}$ is further discretized to the nearest multiple of 10. $TT_{t}$ denotes transition time left, i.e., how many time slots are needed to finish transition to wake or sleep state. $LA_{t}$ is the last applied action. During transitions, the agent does not take new actions to evaluate the effects of the previous one. The rationale behind this state representation is that the agent must observe the current workload and node distribution for each state to determine whether the transition between states is needed, while the transition time left and last action applied determine whether that transition is allowed. Altogether, these parameters under state definition facilitate adaptive changes in the dynamic fog–cloud environment.

In the time slot t and state $S_{t}$, the agent makes the decision and takes an action $A_{t}$ from the predefined discrete action space $A_{t}\in \left\{-TN_{\max},\ldots ,0,\ldots +TN_{\max}\right\}$. The action corresponds to either increasing or decreasing a number of wake nodes by a fixed percent of total computing capacities or to maintaining current node distribution without changes. When the agent applies action $A_{t}=0$, it means that there is no change in node distribution. To ensure efficient system operation, we assume that $WN_{t}\leq WN_{\min}$. The agent adjusts $WN_{t}$ using variable step sizes expressed as multiples of 10%, where the selected step depends on the gap between the current proportion of wake nodes and the discretized demand level. To maintain stability and prevent excessive fluctuations, we define the parameter ${TN}_{\max}$ as the maximum allowed percentage of nodes that transition between states during a single time slot. Therefore, it applies $TN_{t}^{s}\leq TN_{\max}$ and $TN_{t}^{w}\leq TN_{\max}$, where $TN_{t}^{s}$ and $TN_{t}^{w}$ denote the percentage of nodes that transition to sleep and wake states, respectively. These actions directly affect node allocation between energy states and are crucial for compromising energy efficiency and responsiveness of the fog–cloud system.

A reward function $R_{t}$ is obtained after executing action $A_{t}$ in state $S_{t}$. It can be determined based on demand satisfaction and energy consumption. The demand satisfaction component favors matching wake resources and the demand, while the energy consumption component aims to minimize total energy consumption. Since transitions increase energy consumption and nodes respond with delay, the agent may prefer to maintain a steady state rather than initiate a transition, even if it could enhance performance. To prevent the agent from keeping the system unchanged when needed, we introduce a penalty. Moreover, we incentivize the agent to react to the possible burst in demand. Additionally, we reward the agent with $R_{t}^{p}\in \left(0,1\right)$ if it cannot improve the state regardless of the action to be taken. This situation occurs if the demand is lower than the minimum required wake nodes capacity, so the agent cannot decrease wake nodes to sleep, or the demand exceeds the total capacity of fog nodes, so it is impossible to wake more nodes. Therefore, the reward function can be formulated as follows:

(12) $$R_{t}=\rho \left(R_{t}^{demand}+P_{t}+R_{t}^{burst}+R_{t}^{p}\right)-\left(1-\rho \right)R_{t}^{energy},$$

where $\rho \in \left(0,1\right)$ denotes the weighting parameter for the demand satisfaction-energy trade-off; $R_{t}^{demand}$ represents a reward for matching demand and wake fog resources; $P_{t}$ is the penalty segment of the reward; $R_{t}^{burst}$ is a reward for responding during burst; $R_{t}^{p}$ is a reward assigned when the agent cannot improve the state due to system constraints (for example, when demand is below the minimum wake-node threshold or exceeds total fog capacity); $R_{t}^{energy}$ corresponds to the energy-consumption component. Each component contributes a distinct behavioral signal to the agent. The demand-matching term supports maintaining sufficient active capacity; the penalty and burst-handling terms prevent the agent from remaining in a suboptimal state and enable prompt response under sudden burst in traffic arrival, respectively; the energy consumption component promotes long-term efficiency by preventing unnecessary transitions. A linear weighting structure is adopted for interpretability and stable learning behavior, providing a transparent and tunable mechanism for balancing demand satisfaction and energy savings through the parameter ρ.

$R_{t}^{demand}$ can be calculated as

(13) $$R_{t}^{demand}=\left\{\begin{array}{cc} r_{1}, & \Delta_{t}\leq z_{1}\\ r_{2}, & z_{1}<\Delta_{t}\leq z_{2}\\ \vdots & \\ r_{\upsilon -1}, & z_{\upsilon -2}<\Delta_{t}\leq z_{\upsilon -1}\\ r_{\upsilon }, & \Delta_{t}>z_{\upsilon -1} \end{array}\right..$$

In (13), $\Delta_{t}=\left|WN_{t}-D_{t}\right|$ is an absolute mismatch between the demand and wake fog resources, υ denotes the number of reward levels, $z_{\nu }$ represents the increasing mismatch thresholds, and $r_{v},\;v\in \left[1,\upsilon \right]$ represents the non-increasing reward for the demand matching component of the reward function.

The penalty segment of the reward function can be defined as

(14) $$P_{t}=\left\{\begin{array}{cc} -\chi , & \;a_{t}=0\wedge \Delta_{t}>\delta \\ \;\;0, & otherwise \end{array}\right.,$$

where $\chi \in \left(0,1\right)$ denotes the penalty value for not taking actions when the demand gap exceeds the demand mismatch tolerance δ.

To encourage the agent to take an action as a response to possible burst in the demand, we introduce

(15) $$R_{t}^{burst}=\left\{\begin{array}{cc} r^{burst}, & a_{t}\neq 0\wedge \Delta_{t}>\mu \\ \;0, & otherwise \end{array}\right.,$$

where $r^{burst}\in \left(0,1\right)$ is a constant reward for burst handling, while μ denotes the threshold for burst detection.

In (12), the parameter $R_{t}^{p}$ can be expressed as follows:

(16) $$R_{t}^{p}=\left\{\begin{array}{cc} r_{1}, & D_{t}\leq WN_{\min}\wedge \;\;WN_{t}=WN_{\min}\\ r_{1}, & D_{t}\geq 100\wedge \;WN_{t}=100\\ \;0, & otherwise \end{array}\right..$$

Similarly to (13), $r_{1}$ in (16) denotes the maximum reward that agent can get.

The energy component of the reward function normalizes total energy consumption from the objective function (1). For simplicity, we assume that each active node has maximal utilization of resources. Thus, the energy reward can be expressed as

(17) $$R_{t}^{energy}=\frac{E_{t}}{E_{\max}},$$

where $E_{t}$ denotes the total energy consumed:

(18) $$E_{t}=\left(\eta_{t}^{WN}-\eta_{t}^{IN}\right)\cdot e_{peak}+\eta_{t}^{IN}\cdot e_{idle}+\eta_{t}^{SN}\cdot e_{sleep}+\eta_{t}^{T_{w}}\cdot e_{sw}+\eta_{t}^{T_{s}}\cdot e_{ws}$$

In (16), the number of nodes in wake, idle, sleep, transitioning to wake, and transitioning to sleep are denoted as $\eta_{t}^{WN}$, $\eta_{t}^{IN}$, $\eta_{t}^{SN}$, $\eta_{t}^{T_{w}}$ and $\eta_{t}^{T_{s}}$, respectively. These values can be easily derived from the current state and action applied. $E_{t}$ is normalized by the maximum energy that can be consumed by all fog nodes, $E_{\max}=M\cdot e_{peak}$, where M is the number of fog nodes in a fog network.

The AQETO framework employs Q-learning over E episodes to determine the optimal decision-making policy based on the action–value function $Q\left(S_{t},A_{t}\right)$. The agent aims to maximize the cumulative reward by updating the Q-values according to the Bellman equation [54]:

(19) $$Q\left(S_{t},A_{t}\right)\leftarrow Q\left(S_{t},A_{t}\right)+\alpha \left[R_{t}+\gamma \underset{A_{t+1}}{\max}Q\left(S_{t+1},A_{t+1}\right)-Q\left(S_{t},A_{t}\right)\right],$$

where α is a learning rate, and γ is a discount factor, which controls the importance of future rewards. Initially, all Q-values are set to zero for each time slot. At each step, the agent observes the current state $S_{t}$, selects an action $A_{t}$ based on an ε-greedy policy, receives a reward $R_{t}$, and transitions to the next state $S_{t+1}$. The Q-value is then updated accordingly. The ε-greedy policy ensures that the agent chooses the best-known action with probability $\left(1-\varepsilon \right)$, and explores a random action with probability ε. We assume that over time, the value of ε gradually decreases, according to a decay schedule, to encourage more exploitation as learning progresses. Specifically, after each episode, ε is updated as $\varepsilon =\max\left(\varepsilon_{\min},\;\varepsilon \cdot \varepsilon_{decay}\right)$, ensuring that the exploration rate never falls below a defined minimum threshold $\varepsilon_{\min}$. This process continues iteratively, and the average reward is computed over time. Algorithm 1 shows the pseudocode for the Q-learning-based energy consumption management, while Algorithm 2 and Algorithm 3 show the valid action determination and the reward function, respectively.

Algorithm 1 begins by initializing the Q-table for all states and actions (line 1). Afterwards, in line 2, it iterates through all training episodes. Lines 3 and 4 determine the initial setup for each episode, i.e., the distribution of wake, idle, and sleep nodes, the transition counters, the last action applied, and the total reward. For each time slot, the total demand is computed (line 5), discretized (line 6), and used to calculate the percentages of wake, idle, sleep, and transitioning nodes (line 7). These values determine the current state in line 8. Using the ε greedy policy, the agent decides whether to explore one of the available actions (lines 9–11) or select the action with the highest Q-value (line 12). If no transition is active and the chosen action modifies the system state, a new transition is initiated and its duration recorded in lines 13–15. After applying the selected action, the algorithm updates the distribution of wake, idle, and sleep nodes in line 16 and then updates the nodes that are currently transitioning between states in line 17. The reward for the action is computed in line 18, and the next state is determined in line 19. In line 20, the cumulative episode reward is updated, and after each episode, the exploration rate is reduced according to the decay schedule (line 22).

Algorithm 2 determines the valid actions for the current decision step. It begins by reading the current state (line 1) and initializing the set of admissible actions (line 2). If a transition is already in progress (line 3), the remaining transition time is decremented (line 4) and only the action with no change in the node distribution is permitted (lines 5–7). When no transition is active, all actions that modify node distribution are evaluated (line 8). For each candidate action, the magnitude of the required node transitions is calculated in line 9, and the action is checked against system constraints, including the minimum wake-node requirement, the maximum transition magnitude, and bounds on the proportions of idle and sleep nodes (line 10). Feasible actions are added to the valid set in lines 11–13. Finally, the action with no change in the node distribution is included to allow the agent to maintain the current configuration when appropriate (line 15), and the set of valid actions is returned in line 16.

Algorithm 3 computes the reward for a selected action. It initializes all reward components in line 1 and computes the mismatch between the demand and the available capacity of wake nodes in line 2. The reward component that the agent receives if the current state cannot be improved regardless of the action to be taken is determined using Equation (16) in line 3. The algorithm then assigns the demand-matching reward by checking mismatch thresholds (lines 4–9). A penalty is applied if the agent chooses the no-change action while the mismatch exceeds the allowed tolerance (lines 10–14). If a sudden increase in demand is detected and the agent responds with an action that modifies the system state, a burst-handling reward is awarded (lines 15–19). Energy consumption is computed using Equation (18) in line 20 and normalized in line 21. The total reward is then calculated from all components according to Equation (12) in line 22 and returned in line 23.
**Algorithm 1.** AQETO: Energy Management**1**  Initialize Q-table: $Q\left(S_{t},A_{t}\right)$$\;\mathrm{for}\;\mathrm{all}\;S_{t}$$\;\mathrm{and}\;A_{t}$ **2**  **for** each episode = 1 to E **do**
$3\;\;\;\;\;\;WN_{0}\leftarrow 100$$,\;IN_{0}\leftarrow 100$$,\;SN_{0}\leftarrow 0$$,\;TT_{0}\leftarrow 0$, $\;\;\;\;\;LA_{0}\leftarrow 0$, total_reward←0
4      for each time slot t=1toT **do**
$5\;\;\;\;\;\;\;\mathrm{Compute}\;\mathrm{total}\;\mathrm{demand}\;D_{t}$
$6\;\;\;\;\;\;\;\mathrm{Discretize}\;D_{t}$
$7\;\;\;\;\;\;\;\mathrm{Compute}\;WN_{t}$$,\;IN_{t}$$,\;SN_{t}$$,\;TN_{t}^{s}$$,\;TN_{t}^{w}$ $8\;\;\;\;\;\;\mathrm{Determine}\;\mathrm{current}\;\mathrm{state}\;S_{t}\leftarrow \left[D_{t},WN_{t},IN_{t},SN_{t},TT_{t},LA_{t}\right]$ $9\;\;\;\;\;\;\mathrm{Select}\;A_{t}$ using ε-greedy policy:
**10**        with probability ε: 
$\;\;\;\;\;\;\;\;\;\;\mathrm{choose}\;A_{t}\leftarrow$ random valid action using Algorithm 2
**11**        **else**
$12\;\;\;\;\;\;\;\;\;\;\mathrm{choose}\;A_{t}\leftarrow \arg\max_{a\in A_{t}}Q\left(S_{t},a\right)$
**13**       **if**
$TT_{t}=0$$\;\mathrm{and}\;A_{t}\neq 0$ **do**
$14\;\;\;\;\;\;\;TT_{t}\leftarrow \tau \left(A_{t}\right)$$,\;LA_{0}\leftarrow A_{t}$
**15**       **end**
**16**       Apply $A_{t}$$:\;\mathrm{adjust}\;WN_{t}$$,\;IN_{t}$$,\;SN_{t}$ **17**       Update node counts based on transitions
**18**       Compute reward $R_{t}$ using Algorithm 3
**19**       Determine new state $S_{t+1}$ using Equation (16)
**20**       $\text{total\_reward}\leftarrow \text{total\_reward}\;+\;R_{t}$ **21**     **end**
**22**     Update  $\varepsilon \leftarrow \max\left(\varepsilon_{\min},\;\varepsilon \cdot \varepsilon_{decay}\right)$
**23**  **end**

**Algorithm 2.** AQETO: Valid actions$1\;\mathrm{Input}\;\mathrm{current}\;\mathrm{state}\;S_{t}$ $2\;A_{t}\leftarrow \emptyset$ $3\;\;\mathrm{if}\;TT_{t}>0$ **then** $4\;\;\;\;TT_{t}\leftarrow TT_{t}-1$ $5\;\;\;\;\mathrm{set}\;\mathrm{valid}\;\mathrm{actions}:\;A_{t}\leftarrow 0$ $6\;\;\;\;\mathrm{return}\;A_{t}$ **7 end** $8\;\mathrm{for}\;\mathrm{each}\;\mathrm{action}\;a\in A\backslash \left\{0\right\}$ **do** **9**    Determine transition magnitude **10  if** applying *a* satisfies constraints:
$\;\;\;\;WN_{t}\leq WN_{\min}$, 
$\;\;\;\;TN_{t}^{s}\leq TN_{\max},\;TN_{t}^{w}\leq TN_{\max}$     bounds on idle and sleep proportion
**11  then**
**12**   add *a* to $A_{t}$
**13**  **end**
**14**  **end**
$15\;\;\mathrm{add}\;0\;\mathrm{to}\;A_{t}$ $16\;\;\mathrm{return}\;A_{t}$

**Algorithm 3.** AQETO: Reward function$1\;\;\mathrm{Initialize}\;R_{t}^{demand}\leftarrow 0$$,\;R_{t}^{energy}\leftarrow 0$$,\;P_{t}\leftarrow 0$$,\;R_{t}^{burst}\leftarrow 0$$,\;R_{t}^{p}\leftarrow 0$$,\;R_{t}\leftarrow 0$ $2\;\;\mathrm{Compute}\;\mathrm{demand}\;\mathrm{mismatch}\;\Delta_{t}$ **3**  Compute $R_{t}^{p}$ using Equation (16)
4   for i=0 to υ
**do**
$5\;\;\;\;\mathrm{if}\;\Delta_{t}\leq z_{i+1}$ **then** $6\;\;\;\;\;R_{t}^{demand}\leftarrow$
$r_{i}$
**7      break**
**8     end**
**9  end**
$10\;\;\mathrm{if}\;A_{t}=0\;\wedge \;\Delta_{t}>\delta$ **then**
$11\;\;P_{t}\leftarrow -\chi$ **12  else**
$13\;\;P_{t}\leftarrow 0$ **14  end** $15\;\;\mathrm{if}\;\text{burst\_flag}=\mathrm{True}\;\wedge \;A_{t}\neq 0$ **then** $16\;\;R_{t}^{burst}\leftarrow r^{burst}$
**17  else**
$18\;\;R_{t}^{burst}\leftarrow 0$
**19**  **end**
**20**  Compute energy consumption using Equation (18)
**21**  Normalize energy consumption using Equation (17)
**22**  Compute total reward using Equation (12)
**23**  **return** *R_t_*

### 4.2. Task Assignment

In the task assignment phase, the optimal node distribution policy for a given fog–cloud environment is applied, and tasks are allocated to the currently wake nodes, as shown in Algorithm 4. The learned AQETO policy is loaded at the beginning (line 1), and the current state of each fog node is determined in lines 2–4. For each request, the feasible set of fog nodes is determined in lines 6–11, while the cloud feasibility is checked in lines 12–14. The feasible nodes for each request are sorted by their communication delay (line 15), and all tasks are sorted in increasing order of their deadlines (line 17). The updated fog node distribution obtained from the energy-management policy is determined in lines 18–20. Each request is then assigned to the first feasible fog node that is active or idle and has sufficient available capacity (lines 21–28), and the node’s remaining capacity is updated accordingly. If no fog node can serve the request, cloud assignment is attempted if it meets the deadline constraint (lines 29–33).
**Algorithm 4.** AQETO: Task assignement**1** Load saved AQETO policy (Algorithm 1) $2\;\mathrm{for}\;\mathrm{each}\;f_{j}\in F$ **do** **3**   determine current node state **4** **end** $5\;\mathrm{for}\;\mathrm{each}\;rq_{i}\in RQ$ **do** $6\;\;\;\Phi_{i}\leftarrow \emptyset$ $7\;\;\;\mathrm{for}\;\mathrm{each}\;f_{j}\in F$ **do** $8\;\;\;\;\mathrm{Take}\;l_{ij}$ from pre-computed all-pair shortest paths
$9\;\;\;\;\mathrm{if}\;l_{ij}\leq d_{i}$ **then**
$10\;\;\;\;\;\;\Phi_{i}\leftarrow \Phi_{i}\cup \left\{f_{j}\right\}$
**11**      **end**
$12\;\;\mathrm{if}\;l_{ic}\leq d_{i}$ **do**
$13\;\;\;\Phi_{i}\leftarrow \Phi_{i}\cup \left\{c\right\}$
**14**  **end**
$15\;\;\mathrm{Sort}\;\Phi_{i}$ in non-increasing order per minimum delay **16**  **end**
17  Sort all requests in RQ in increasing order per their maximum delay tolerance $18\;\;\mathrm{for}\;\mathrm{each}\;f_{j}\in F$ **do**
**19**  determine novel node state **20**  **end**
$21\;\;\mathrm{for}\;rq_{i}\in RQ$ **do** $22\;\;\mathrm{for}\;f_{j}\in \Phi_{i}$ **do** $23\;\;\;\mathrm{if}\;f_{j}$ in active or idle state **do** $24\;\;\;\;\;\;\mathrm{if}\;\mathrm{available}\;\mathrm{capacity}\;\geq \sigma_{i}$ **do**
**25**        $f_{j}$ state = active
**26**        assigned = True
**27**        update $f_{j}$ available capacity
**28**        **break**
**29**  **if** not assigned **do**
$30\;\;\;\mathrm{if}\;c\in \Phi_{i}$ **do**
**31**      assigned  = True
**32**   **end**
**33**  **end**
**34**  **end**

## 5. Performance Evaluation

In this section, we describe the extensive simulations performed to evaluate the performance of the proposed AQETO framework. All simulations are conducted in Python 3.10.11 on a Windows 11 Pro PC with an AMD Ryzen 3 GHz CPU and 16 GB of memory.

### 5.1. Experimental Setup

Since no single study comprises all network, delay, energy, and task-related parameters together, some values used in our experimental setup are drawn from representative sources that provide realistic and compatible ranges for each parameter type. However, some parameters are modeling choices intended to provide a consistent and controlled evaluation environment for AQETO. It should be noted that AQETO does not rely on these specific numerical settings. In practice, the framework can be configured using the actual delay, workload, and energy characteristics of the deployed fog–cloud environment.

In terms of environment settings, we assume there is a single cloud data center in the cloud layer [48]. The fog layer is represented as a network of fog nodes, where the number of fog nodes varies between 10 and 30. The computation capacity of each fog node is 100 CRBs. We randomly generated fog network topology by the Watts-Strogatz small-world graph model [55], with parameters k=4 and p=0.2. The average number of IoT devices ranges between 50 and 150. Parameter values for network settings are in accordance with [4]. Therefore, link weights in the fog network are randomly assigned in [3, 5] ms, while links between fog nodes and a cloud data center are randomly assigned in [140, 160] ms. Link weights between IoT devices and fog nodes range in [10, 15] ms. We randomly generate 100 instances with 100 time slots for each network setting. The duration of each time slot Δt=1 s [56]. At the beginning of the time slot, fog nodes defined as wake using the AQETO energy management policy in the previous time slot are randomly declared as active by assigning s workload in range of 1–100 CRBs, while the rest of the wake nodes (if any) are declared as idle.

Energy consumption is expressed in Energy Units (EUs), which are abstract, dimensionless values used exclusively for the purpose of comparative evaluation of the offloading algorithms. EUs are not physical units and do not model hardware-specific energy consumption. Instead, they are used to consistently quantify relative differences between the fog node energy states. Following the qualitative behavior of multi-sleep-mode servers in [53], energy consumed at peak load, in idle, and sleep state are set at 100 EUs, 60 EUs, and 30 EUs, respectively, while transitions from wake to sleep and from sleep to wake consume 20 EUs and 40 EUs, respectively. Transition durations from wake to sleep and sleep to wake states are one and two time slots, respectively. Although the exact numeric values differ from those in [53], they follow the same proportional ordering, thus ensuring that AQETO operates under realistic dynamics. We assume that at least 30% of fog nodes must be awake in each time interval, i.e., $WN_{\min}=30$. Requests for task offloading are generated at the beginning of each time slot. The maximum delay tolerance and size are randomly assigned to each task in the range [1, 4] s, according to [57], and [2, 3] CBRs, according to [51], respectively. To validate the effectiveness of the AQETO framework, we analyze two scenarios with different task arrival patterns: (i) Poisson arrival, with the parameter λ=1; (ii) bursty task arrival, where T time slots are divided into 10 frames of 10 time slots (in each time frame, the arrival rate of the first 4 time slots is 5 times greater than in the remaining 6 time slots [56]. These two patterns reflect common IoT traffic dynamics, with Poisson arrivals modeling regular load and bursty arrivals modeling event-driven load fluctuations [58].

All reinforcement learning-based parameters used in the simulation are presented in Table 2. The Q-learning parameters (number of episodes, learning rate, discount factor, and exploration schedule) follow commonly adopted ranges in reinforcement learning practice. The remaining reward-related parameters are tuned empirically through preliminary tests to ensure that each reward component contributes meaningfully to the learning process.

To the best of our knowledge, this is the first paper that uses reinforcement learning to minimize energy consumption of fog nodes by minimizing the number of idle fog nodes and jointly minimize delay in the task offloading process. Therefore, there is no directly comparable approach to the proposed AQETO framework. To evaluate the effectiveness of AQETO, we developed 4 baseline offloading algorithms:

1. Minimum Active Nodes Task Offloading (MANTO): Tasks are sorted by urgency. Each task is assigned to the feasible active fog node with the maximum load to minimize the number of active nodes or to a feasible idle fog node. Remaining nodes stay in an idle state. When fog computing resources are exceeded, the task is assigned to the cloud (if feasible by the deadline).
2. Minimum Active Nodes Task Offloading with Sleep Nodes (MANTO_SL): This algorithm follows the same task selection and offloading principle of MANTO. However, remaining idle nodes are switched to sleep state to reduce energy consumption. It should be noted that this algorithm assumes that nodes are immediately switched between states; it neglects transition durations and additional energy consumption due to the transition. With this simplified design, MANTO_SL represents an optimistic lower-bound case in terms of energy consumption, in which transitions between energy states occur instantaneously.
3. Greedy (GR): Tasks are sorted by the urgency. Each task is assigned to the feasible fog node with minimum delay.
4. Random (RA): Tasks are randomly selected and randomly assigned to the feasible fog nodes. Once fog computing resources are exceeded, tasks are assigned to the cloud when feasible.

### 5.2. Numerical Results

We evaluate the performance of the proposed AQETO framework and compare it against the baseline algorithms in both traffic arrival scenarios. Initially, the convergence analysis of AQETO is provided. Afterwards, we use several evaluation criteria for comparison, including average energy consumption per time slot, average delay, system efficiency, and cloud allocation ratio.

#### 5.2.1. Convergence Analysis

**Figure 3:**
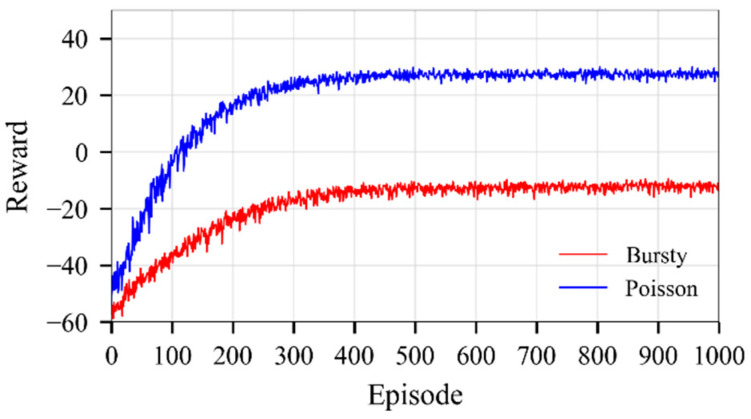
Convergence curves, *m* = 10, *n* = 100.

Figure 3 shows the convergence curves for both scenarios when the average number of requests for task offloading is 100, and there are 10 fog nodes in the fog network. The proposed model converges after 300–400 episodes under both traffic arrival patterns. However, the Poisson traffic arrival pattern converges faster and achieves higher average rewards. The model also stabilizes under bursty traffic conditions, but with lower average reward due to significant fluctuations in demand for fog computing resources. Similar results apply for all other network configurations.

#### 5.2.2. Energy Consumption Analysis

**Figure 4:**
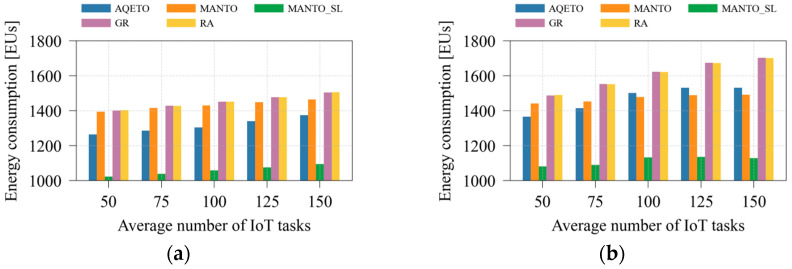
Energy consumption under different task offloading algorithms, where *m* = 20: (**a**) the Poisson task arrival pattern; (**b**) Bursty task arrival pattern.

Figure 4 illustrates the total energy consumption as the average number of IoT tasks varies under different task offloading algorithms and both task arrival scenarios. Compared with the baseline algorithms that do not aim to minimize the number of active fog nodes (GR and RA), the proposed AQETO achieves an average energy saving of approximately 10% under both Poisson and bursty task arrival patterns. The MANTO algorithm, which minimizes the number of active fog nodes but keeps inactive ones idle, provides modest energy savings under the Poisson scenario, while the reductions become more pronounced under bursty arrivals. As shown in Figure 4b, the bursty scenario poses greater challenges for AQETO, as it struggles to outperform MANTO in terms of energy consumption when the average number of IoT tasks increases. The behavior occurs because MANTO adjusts the number of active nodes without considering transition delays, thereby achieving lower energy consumption when the average number of IoT tasks increases. On the other hand, AQETO considers transition duration and node unavailability during transition. Concurrently, it aims to minimize frequent switching between the states. These factors constrain AQETO’s responsiveness during rapidly fluctuating workloads, but they ensure long-term feasibility at the cost of slightly higher energy consumption. Among all compared schemes, apart from AQETO, only the MANTO_SL algorithm introduces fog node switching into a sleep energy mode. This approach achieves the greatest reduction in total energy consumption under both task arrival scenarios. However, as emphasized earlier, MANTO_SL neglects fog node transition delays and node unavailability during transitions. Therefore, it serves primarily as an indicator of how transition effects influence overall energy consumption.

**Figure 5:**
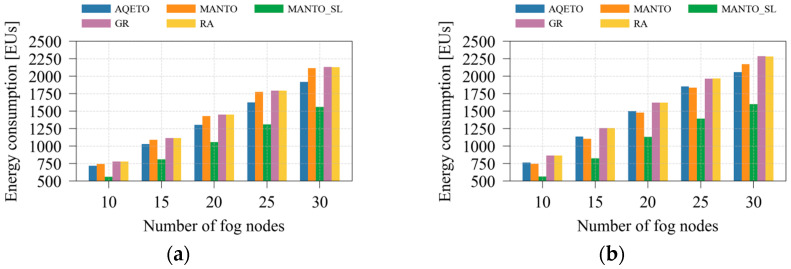
Energy consumption under different task offloading algorithms, where *n* = 100: (**a**) the Poisson task arrival pattern; (**b**) Bursty task arrival pattern.

The impact of varying the number of fog nodes on total energy consumption is presented in Figure 5 for both Poisson and bursty task arrival scenarios. In this case, the bursty scenario leads to only a negligible increase in total energy consumption. Among the compared algorithms, MANTO_SL achieves the highest energy savings, while the energy reduction achieved by AQETO relative to MANTO slightly increases with a larger number of fog nodes in the system.

#### 5.2.3. Delay Performance Analysis

**Figure 6:**
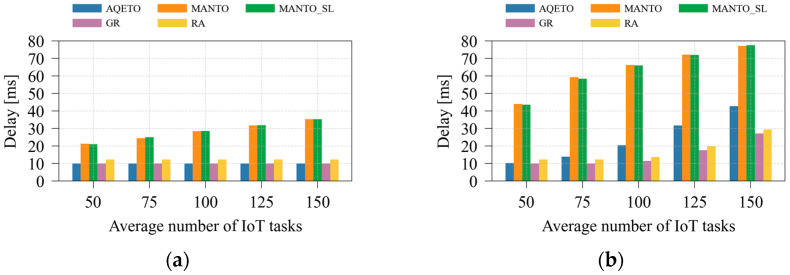
Average delay under different task offloading algorithms, where *m* = 20: (**a**) the Poisson task arrival pattern; (**b**) Bursty task arrival pattern.

**Figure 7:**
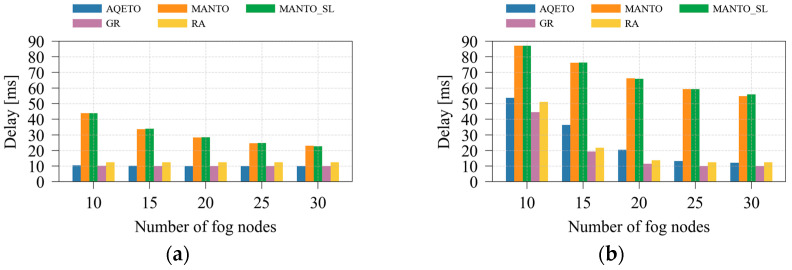
Average delay under different task offloading algorithms, where *n* = 100: (**a**) the Poisson task arrival pattern; (**b**) Bursty task arrival pattern.

Figure 6 and Figure 7 illustrate the effects of varying the average number of IoT tasks and the number of fog nodes on task offloading delay under both task arrival scenarios. As expected, the bursty task arrival pattern significantly increases delay, particularly for the MANTO and MANTO_SL algorithms. These algorithms primarily aim to minimize energy consumption, with delay reduction addressed only indirectly through the prioritization of the most urgent tasks. Consequently, under both arrival scenarios, MANTO and MANTO_SL exhibit the highest delay values. The GR algorithm, in contrast, explicitly prioritizes urgent tasks and assigns them to the most suitable fog nodes with minimum expected delay, making it the baseline benchmark for delay minimization. However, GR disregards energy-consumption considerations. Similarly, the RA algorithm neglects both energy and delay factors, which results in longer delays compared with GR. In some scenarios with a greater number of IoT tasks, RA may achieve competitive delay performance or even outperform AQETO slightly. However, this result is not driven by a delay optimization policy but by random task assignment at the cost of increased energy consumption. The proposed AQETO algorithm achieves delay performance comparable to that of GR, maintaining low latency while balancing energy efficiency. Nevertheless, its performance slightly deteriorates under bursty task arrivals and higher numbers of IoT tasks.

An increase in the number of fog nodes contributes to a significant reduction in task-offloading delay, particularly under the bursty task arrival scenario, as depicted in Figure 7. This behavior can be attributed to the improved resource availability and load distribution across the expanded fog infrastructure. The proposed AQETO algorithm consistently achieves the minimum delay under both Poisson and bursty arrivals, demonstrating its effectiveness in exploiting larger fog networks.

#### 5.2.4. System Efficiency

In this paper, system efficiency is quantified by the success rate, defined as the ratio of successfully completed task offloading requests to the total number of requests. A task is considered successfully completed if and only if it is finished within its deadline, regardless of whether it is executed at a fog node or in the cloud. Although the cloud is assumed to have sufficient computational capacity, the communication delay to the cloud is significantly higher than in the fog layer, making cloud offloading infeasible for many urgent tasks, particularly under the bursty traffic scenario. Consequently, not all task offloading requests can be completed before their deadlines, and the system efficiency does not always reach 100%.

**Figure 8:**
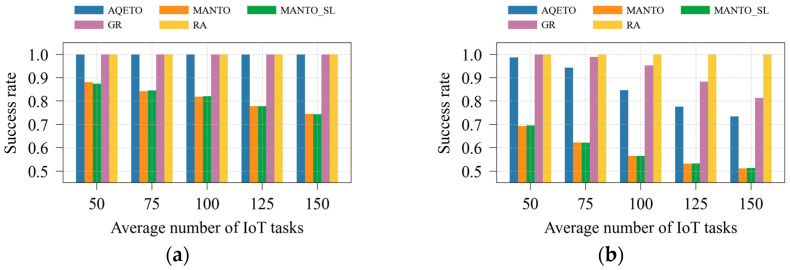
Success rate under different task offloading algorithms, where *m* = 20: (**a**) the Poisson task arrival pattern; (**b**) Bursty task arrival pattern.

**Figure 9:**
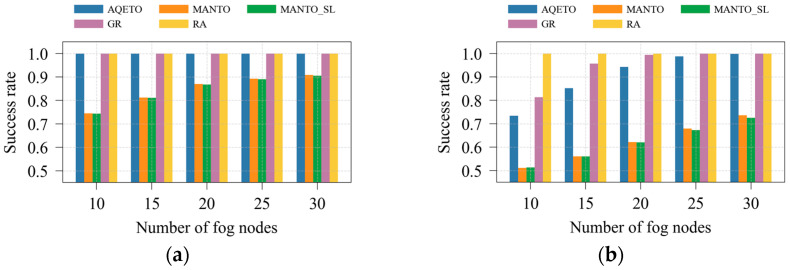
Success rate under different task offloading algorithms, where *n* = 100: (**a**) the Poisson task arrival pattern; (**b**) Bursty task arrival pattern.

As shown in Figure 8a and Figure 9a, the proposed AQETO algorithm attains the highest success rate under the Poisson task arrival scenario, comparable to the GR and RA algorithms. Under the bursty arrival scenario, illustrated in Figure 8b and Figure 9b, the success rate remains above 70%, demonstrating the robustness of AQETO in handling fluctuating workloads. In contrast, the MANTO and MANTO_SL algorithms exhibit the lowest success rates across both task arrival scenarios.

#### 5.2.5. Cloud Allocation Ratio

AQETO assumes that task allocation to the cloud should occur only when necessary, given the additional delay introduced by cloud processing. Consequently, cloud offloading appears primarily under the bursty task arrival scenario and increases with the average number of IoT tasks, as shown in Table 3. In contrast, the GR and RA algorithms do not aim to minimize the number of active fog nodes. Therefore, their cloud allocation ratios remain very low, even under bursty conditions (below 12.3%). On the other hand, MANTO and MANTO_SL focus on minimizing energy consumption at the fog layer, which leads to a substantial increase in cloud offloading, reaching up to 46%.

## 6. Discussion

The comparative analysis demonstrates that the proposed AQETO algorithm effectively balances the trade-offs among energy consumption, delay, success rate, and cloud offloading in a fog environment. By adaptively managing resource allocation, AQETO minimizes energy consumption while maintaining acceptable performance in terms of delay and success rate. It consistently consumes less energy than non-energy-aware approaches such as GR and RA, while avoiding the substantial delay increase observed in energy-oriented algorithms like MANTO and MANTO_SL. It should be noted that MANTO_SL represents an ideal lower-bound in terms of energy consumption, as it assumes instantaneous transitions between energy states and neglects transition delays and resource unavailability during transitions. This advantage becomes particularly prominent under bursty traffic arrival scenario. However, AQETO models realistic constraints, such as transition durations, resource unavailability and additional transition overhead, which results in slightly increased energy consumption. Despite RA can achieve competitive delay in some scenarios due to random load assignment, it does not address energy consumption. In contrast, AQETO ensures balanced trade-offs across all observed performance metrics.

Furthermore, by limiting unnecessary task migration to the cloud, AQETO preserves high offloading efficiency and reduces external network dependency. These outcomes suggest that intelligent resource control at the fog layer can achieve a favorable balance between energy savings and service quality.

AQETO also maintains an appropriate balance between demand satisfaction and energy conservation. By dynamically adjusting the number of active fog nodes in response to workload intensity, it ensures sufficient computational capacity to handle incoming tasks without excessive power expenditure. This trade-off between service availability and energy efficiency represents a practical and sustainable approach to fog resource management, particularly under fluctuating workloads and time-varying IoT demand.

The results also highlight the importance of real-time, learning-assisted control for sustainable fog operation. Reinforcement-based mechanisms such as AQETO can extend infrastructure lifetime, improve resource utilization, and reduce dependence on cloud resources. Although GR and RA exhibit lower cloud allocation ratios, these algorithms keep all fog nodes always awake. As a result, offloading to the cloud remains low, but only at the expense of substantially higher energy consumption. In contrast, AQETO aims to dynamically adjust the number of active nodes and minimize the number of idle nodes by transitioning them when appropriate. This behavior may lead to a higher cloud offloading ratio under the bursty task arrival scenario, but it remains consistent with AQETO’s objective of improving resource utilization and energy efficiency by avoiding unnecessary transitions between energy states.

Moreover, the findings confirm that minimal energy consumption often entails service degradation, emphasizing the importance of strategies that balance energy reduction with delay and reliability objectives, which is an aspect particularly relevant for delay-sensitive IoT applications.

## 7. Conclusions

This paper proposed AQETO, a reinforcement-based task offloading algorithm for fog environments that dynamically determines the number of active fog nodes, switches idle nodes to sleep mode, and considers transition delays and node unavailability during switching. By adapting the number of active nodes to current demand, AQETO maintains sufficient computational capacity while avoiding unnecessary energy use. The results under both Poisson and bursty task arrival scenarios show that AQETO achieves a balanced performance in terms of energy consumption, delay, success rate, and cloud offloading. It reduces total energy consumption compared with non-energy-aware algorithms while keeping delay and success rate at competitive levels. By considering the temporary unavailability of nodes during transitions, it ensures smooth operation and continuous service even under varying workloads. Moreover, by limiting unnecessary offloading to the cloud, AQETO decreases network dependency and improves overall system efficiency.

Overall, AQETO demonstrates that reinforcement-based control can successfully balance energy savings and performance in fog–IoT systems. Its adaptive management of fog node states provides a simple yet effective way to improve energy efficiency without compromising responsiveness. As future work, we will focus on extending AQETO to heterogeneous fog infrastructures with varying node capacities and energy characteristics. A promising extension could be addressing inter-task dependencies or workflow-based tasks. In addition, we plan to explore predictive scheduling of state transitions to anticipate workload variations and further reduce unnecessary switching. Another important direction will be to integrate cooperative or multi-agent decision-making among distributed fog nodes.

## Figures and Tables

**Table 1:** List of notations and corresponding descriptions.

Notation	Description
Δt	The length of a time slot
T	The number of time slots
E	The number of training episodes
RQ	Set of all task offloading requests
n	Number of task offloading requests
$d_{i}$	The minimum delay tolerance
$\sigma_{i}$	Task size (in CRBs)
F	Set of all fog nodes
m	Number of fog nodes
$\theta_{j}$	Computing capacity of a fog node (in CRBs)
$l_{ij}$	The shortest delay to a fog node
$l_{ic}$	The shortest delay to the cloud
$\Phi_{i}$	$\mathrm{Feasibility}\;\mathrm{set}\;\mathrm{for}\;rq_{i}$
W	Set of wake fog nodes
AC	Set of active fog nodes
I	Set of idle fog nodes
SL	Set of sleep fog nodes
$T_{s}$	Set of fog nodes transitioning to sleep state
$T_{w}$	Set of fog nodes transitioning to wake state
$e_{peak}$	Energy consumed at maximum load
$e_{idle}$	Energy consumed in idle state
$e_{sleep}$	Energy consumed in sleep state
$e_{ws}$	Energy consumed for transitioning to sleep
$e_{sw}$	Energy consumed for transitioning to wake
$\omega_{j}$	Occupied fog node computation resources
$\tau_{s}$	Transition duration to sleep (in time slots)
$\tau_{w}$	Transition duration to wake (in time slots)
$D_{t}$	Discretized demand
$S_{t}$ $,\;A_{t}$	State space, action space
$WN_{t}$	Wake fog nodes’ capacity out of total fog computing capacity (in %)
$IN_{t}$	Idle fog nodes’ capacity out of total fog computing capacity (in %)
$SN_{t}$	Sleep fog nodes’ capacity out of total fog computing capacity (in %)
$TT_{t}$	Transition time left (in time slots)
$LA_{t}$	Last action applied
p	Maximum transition magnitude (in %)
$WN_{\min}$	Minimum wake fog nodes’ capacity out of total fog capacity (in %)
$TN_{t}^{s}$	Fog nodes capacity transitioning to sleep out of total fog capacity (in %)
$TN_{t}^{w}$	Fog nodes capacity transitioning to wake out of total fog capacity (in %)
ρ	Demand–energy trade-off weighting parameter
$R_{t}^{demand}$	Demand satisfaction reward
$\Delta_{t}$	Absolute mismatch between demand and wake fog capacity (in %)
$r_{\nu }$	Reward for demand matching
$z_{\nu }$	Increasing demand mismatch threshold
$P_{t}$	Inaction penalty
χ	Penalty value
δ	Demand mismatch tolerance
$R_{t}^{burst}$	Burst handling reward
$r^{burst}$	Burst reward value
μ	Burst detection threshold
$R_{t}^{p}$	Inaction reward
$R_{t}^{energy}$	Energy consumption minimization reward
$E_{t}$	Total energy consumed
$E_{\max}$	The maximum energy that can be consumed
$\eta_{t}^{WN}$	The number of nodes in wake state
$\eta_{t}^{IN}$	The number of nodes in idle state
$\eta_{t}^{SN}$	The number of nodes in sleep state
$\eta_{t}^{T_{w}}$	The number of nodes transitioning to wake state
$\eta_{t}^{T_{s}}$	The number of nodes transitioning to sleep state
α	Learning rate
ε	Exploration rate
γ	Discount factor
$\varepsilon_{\min}$	The minimum threshold for exploration rate
$\varepsilon_{decay}$	Decay schedule for the exploration rate

**Table 2:** Values of reinforcement learning parameters.

Parameter	Value
The number of episodes, *E*	1000
Learning rate, α	0.7
Discount factor, γ	0.95
Exploration: ε, $\varepsilon_{\min}$, $\varepsilon_{decay}$	1.0, 0.01, 0.99
Demand–energy trade-off weighting parameter, ρ	0.6
Reward for demand matching, $r_{1}-r_{5}$	1.0, 0.01, −0.1, −0.5, −0.7
Demand mismatch thresholds, $z_{1}-z_{4}$	10, 20, 30, 40
Penalty value, χ	0.5
Demand mismatch tolerance, δ	20
Burst detection threshold, μ	40
Burst reward value, $r^{burst}$	0.2

**Table 3:** Cloud allocation ratio (in %), for *m* = 20.

Task Arrival Scenario	Average Number of IoT Tasks	AQETO	MANTO	MANTO_SL	GR	RA
Poisson	50	0.0	6.5	6.2	0.0	0.0
	75	0.0	8.6	9.1	0.0	0.0
	100	0.0	11.5	11.6	0.0	0.0
	125	0.0	13.9	14.0	0.0	0.0
	150	0.0	16.4	16.4	0.0	0.0
Bursty	50	0.1	22.6	22.4	0.0	0.0
	75	2.6	33.6	33.0	0.0	0.0
	100	7.3	38.6	38.4	1.0	1.0
	125	15.2	42.8	42.7	5.2	5.4
	150	22.9	46.3	46.6	12.0	12.3

## Data Availability

The raw data supporting the conclusions of this article will be made available by the authors on request.

## Abbreviations

The following abbreviations are used in this manuscript:

AQETO	Adaptive Q-learning-based Energy-aware Task Offloading
IoT	Internet of Things
ITS	Intelligent Transportation Systems
MSA	Modified Simulated Annealing
OEeRA	Optimal Energy-efficient Resource Allocation
MCRA	Minimal Cost Resource Allocation
FIR	Fault Identification and Rectification
MAB	Multi-Armed Bandit
EDABTOS	Energy-Delay Aware Binary Task Offloading Strategy
DELTA	Deadline-aware Energy and Latency-optimized Task Offloading
MIP	Mixed Integer Programming
MEC	Mobile Edge Computing
SAC	Soft Actor–Critic
SSL	Self-Supervised Learning
VEC	Vehicle Edge Computing
RSUs	Road Side Units
GRPO	Group Relative Policy Optimization
MDP	Markov Decision Process
DTRL	Decision Tree Empowered Reinforcement Learning
DQN	Deep Q-Network
DDQN	Double Deep Q-Network
CADCO	Adaptive Dynamic Cloud–fog Computing Offloading Method for Complex Dependency Tasks
MADRL	Multi-Agent Deep Reinforcement Learning
QoE	Quality of Experience
MATD3-TORA	Multi-Agent Twin Delayed Deep Deterministic Policy Gradient for Task Offloading and Resource Allocation
UAV	Unmanned Aerial Vehicle
MARL	Multi-Agent Reinforcement Learning
GRL	Graph Reinforcement Learning
AFL	Asynchronous Federated Learning
D2D	Device to Device
GNN	Graph Neural Network
QoS	Quality of Service
CRBs	Computing Resource Blocks
SDN	Software Defined Network
EUs	Energy Units
MANTO	Minimum Active Nodes Task Offloading
MANTO_SL	Minimum Active Nodes Task Offloading with Sleep Nodes
GR	Greedy
RA	Random
